# Synergism and Mutualism in Non-Enzymatic RNA Polymerization

**DOI:** 10.3390/life4040598

**Published:** 2014-11-03

**Authors:** Hussein Kaddour, Nita Sahai

**Affiliations:** Department of Polymer Science, The University of Akron, Akron, OH 44325-3909, USA; E-Mail: hkaddour@uakron.edu

**Keywords:** activated nucleotides, peptides, single-chain amphiphile, mineral surfaces, protocell, organocatalysis, RNA polymerization, RNA-peptide world

## Abstract

The link between non-enzymatic RNA polymerization and RNA self-replication is a key step towards the “RNA world” and still far from being solved, despite extensive research. Clay minerals, lipids and, more recently, peptides were found to catalyze the non-enzymatic synthesis of RNA oligomers. Herein, a review of the main models for the formation of the first RNA polymers is presented in such a way as to emphasize the cooperation between life’s building blocks in their emergence and evolution. A logical outcome of the previous results is a combination of these models, in which RNA polymerization might have been catalyzed cooperatively by clays, lipids and peptides in one multi-component prebiotic soup. The resulting RNAs and oligopeptides might have mutualistically evolved towards functional RNAs and catalytic peptides, preceding the first RNA replication, thus supporting an RNA-peptide world. The investigation of such a system is a formidable challenge, given its complexity deriving from a tremendously large number of reactants and innumerable products. A rudimentary experimental design is outlined, which could be used in an initial attempt to study a quaternary component system.

## 1. Introduction

One of the major steps in the RNA world scenario of the origins of life, which remains unsolved, is the transition from random nucleotides to ribozymes capable of self-replication. Recently, major advances in the polymerization of nucleotides in various environments have been reported [1,2,3], but there is still a gap in the pathways linking the products of non-enzymatic polymerization to self-sustained RNA replicases. A review of the literature on this subject reveals a major conceptual limitation in the current studies of prebiotic RNA polymerization, which is the choice of experimental conditions and the likelihood of the availability on early Earth of the reactants used in the polymerization reaction.

The early Earth period relevant to the origins of life extends from the formation of the Earth at 4.56 Ga to ~3.8–3.5 Ga. The latter boundary is based on the discovery of the first body fossils of bacteria in rocks of 3.5 Ga in age [4], although there is some highly debated carbon isotope evidence from metamorphosed sedimentary rocks located in Isua, Greenland, for life at 3.8 Ga [5]. Models of early atmospheric composition, climate and compositions of aqueous reservoirs are still speculative, but it is generally accepted that average environmental conditions were extreme compared to those on current Earth [6,7,8,9,10]. The Sun was a faint star at that time, and heating of Earth’s surface by solar insolation was only about 70% that at present time [11]. By itself, this would suggest low surface temperatures on the Earth with frozen water ice, resulting in a “snowball” Earth, but the presence of greenhouse gases, such as CH_4_, H_2_O and CO_2_, would have contributed to a substantial greenhouse effect. Even in the absence of greenhouse gases, heavy bombardment by bolides may have generated sufficient heat to melt the ice [12]. Heat flow from the Earth’s interior to the surface was significantly higher, ~140 W/m^2^, compared to present day values between 60–100 mW/m^2^ [13]. Oxygen isotopic analyses of the oldest mineral found in Western Australia, a zircon of 4.4 Ga age, indicate that the crystal was formed in equilibrium with liquid water at temperatures of ~80 °C [14]. There is much controversy in the literature about the atmospheric oxygen level and the timing of its increase [15,16], but it is widely accepted that oxygen partial pressure even at 3 Ga was 10,000-times less than present atmospheric levels [17]. It is important to note, however, that these inferences are drawn from a very limited number of rock samples from the Archaean era up to 3.8 Ga. It is, therefore, possible that the environmental conditions recorded in the isotopic and elemental signatures of these rocks represent only the local environments under which they formed. In other words, there may have been microenvironments with freezing temperatures, for example, and others with liquid water at 80 °C. The crust of the Earth also evolved over the relevant period, thus providing a greater diversity of environments for life to emerge [18]. The earliest crust was composed of komatiites, which are Mg-rich basalt-like rocks. The 4.2 Ga Acasta Gneiss in Northwestern Canada represents the first continental-type granitoid-tonalite rocks, and sedimentary rocks of 3.8 Ga age are found in Isua, Greenland. Thus, diverse geochemical environments, such as oceans, deep ocean hydrothermal vents, volcanic crater lakes, fumaroles, intertidal coastal zones, inland evaporite lakes and continental freshwater would have appeared. The chemical compositions of aqueous solutions in these different environments vary tremendously, for example, pH from 0.5–11, ionic strengths from freshwater to hypersaline, and different, major, minor and trace, ion concentrations. Thus, life could have originated in any of these environments, and extant life occupies every environmental niche examined so far.

Experimental models for the bottom-up assembly of a protocell require the consideration of multi-component systems. It is unrealistic to use pure compounds as unique reactants, and minerals that constitute the entire Earth cannot be excluded either. Indeed, clays have been found to enhance self-assembly of biomolecules, such as RNA [19], peptides [20] and lipid molecules [21]. Other prebiotic compounds, though not directly involved in the polymerization reaction, should also be included in the primordial soup, because they might have had essential effects in controlling one or more of the chemical steps, such as pH buffering, displacing an equilibrium, *etc.* [22]. A heterogeneous soup is more prebiotically plausible, but finding the best recipe would be a combinatorial nightmare.

**Figure 1:**
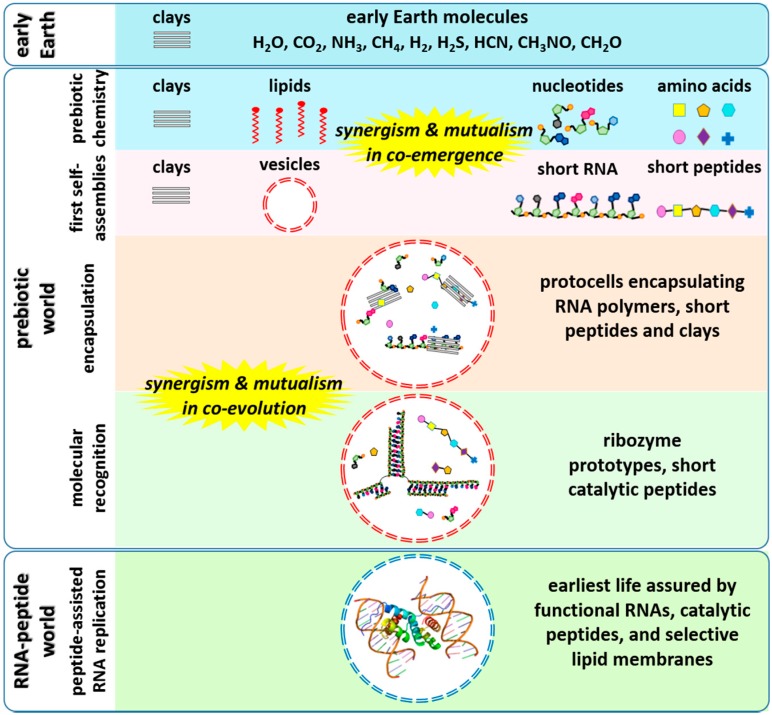
*In vesiculo* chemical evolution of RNA- and protein-like molecules towards an RNA-peptide world. In this scenario, RNAs, peptides and lipids might have co-emerged and co-evolved in a synergistic and mutualistic manner.

At least three different catalysts have been found to enhance the non-enzymatic polymerization of RNA, namely montmorillonite surfaces [19], lipid surfaces [23] and, more recently, a peptide [24]. One question that arises from these findings is what would happen if all of these catalysts (clay, lipid and peptide) were present in the same reaction mixture? From an enzymological standpoint, three cases could arise: synergism, if the combined effect of the catalysts is greater than the addition of the effects of each catalyst alone; antagonism, in which the combined effect of the catalysts is smaller than the addition of the effects of each catalyst alone; or an additive effect, where the combined effect of the catalysts is not significantly different from the addition of the effects of each catalyst alone. Another interesting phenomenon, mutualism, could also occur in such a system. In this effect, RNA oligomers and peptides may have helped each other to evolve towards functional molecules by forming aptamer/metabolite binding complexes (Figure 1). It is also important to note that a completely different perspective on the origins of life holds that energy transduction or metabolism had to precede the synthesis of a molecule as complex as RNA. The hypothesis of co-emergence and co-evolution of RNA and peptides provides a bridge between the RNA-first and metabolism-first perspectives.

In this review, the major focus is on the emergence of the first RNA oligomers. An introductory section to define the main steps of non-enzymatic RNA polymerization and the RNA world hypothesis are described. The methods used for synthesizing activated nucleotides, essential for non-enzymatic RNA polymerization, are summarized. Major advances in non-enzymatic RNA oligomerization and some of their limitations are then presented. Finally, we outline a new model based on a multi-component prebiotic soup.

## 2. Non-Enzymatic RNA Polymerization

**Figure 2:**
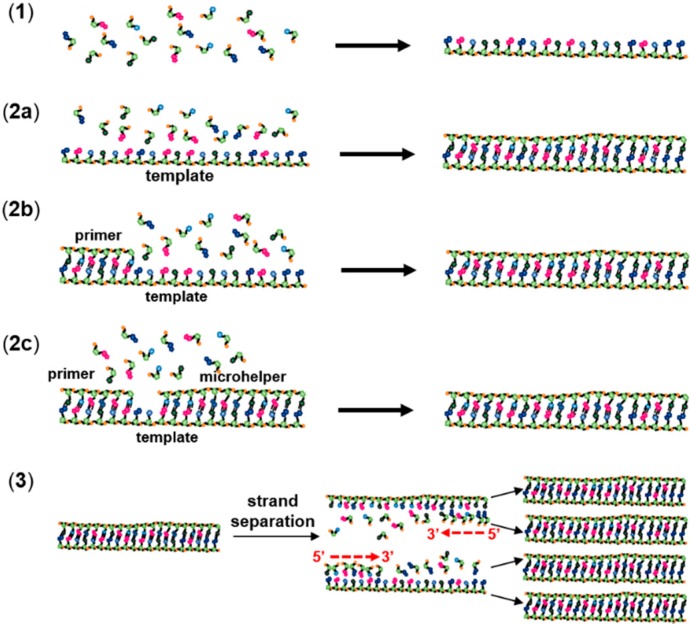
Experimental models of non-enzymatic RNA polymerization. (1) RNA oligomerization; (2a) RNA templation; (2b) template-directed primer extension; (2c) template-directed primer extension in the presence of a microhelper; and (3) non-enzymatic RNA replication.

The process of non-enzymatic polymerization of RNA, as generally accepted in the literature, can be divided into three smaller steps, namely (1) the emergence of the first RNA oligomers, which would have provided the basis for (2) template-directed RNA polymerization, resulting optimistically in robust and functional RNA polymers capable of self-ligation and self-catalysis, finally leading to (3) a non-enzymatic RNA replication (Figure 2). In the present review, the focus was on work in which the first step was studied (1). The term “oligomerization” was used to designate this step, which corresponds to a condensation of RNA monomers in the absence of a template. For template-directed RNA polymerization, three different experiments have been described in the literature: “RNA templation” in the absence of a primer (2a in Figure 2), “RNA primer-extension” in the presence of a primer (2b in Figure 2) and RNA primer extension in the presence of a downstream oligomer (2c in Figure 2). A downstream oligomer, also called a “helper oligonucleotide” [25,26] or microhelper [1], is generally used to assist the incorporation of activated monomers. The microhelper also facilitates the analytical characterization of the product by limiting the incorporation to one or few nucleotides. Finally, “non-enzymatic RNA replication” (3) designates repeating cycles of template-directed RNA polymerization, similar to a polymerase chain reaction (PCR) (Figure 2).

## 3. The RNA World Hypothesis

In the RNA world hypothesis, it is suggested that the earliest cellular life was driven by RNA polymers, which could have simultaneously played the roles of biocatalysts and carriers of the genetic code [27]. To date, there is still no strong evidence for such a statement, but recent advances lend support to the credibility of an antecedent RNA world. For instance, naturally occurring ribozymes in the genomes of certain eukaryotes [28,29,30,31], including human [32,33,34,35] and plants [36], were considered an oddity, but it now appears that ribozymes are widespread among almost all kingdoms of life [37]. These findings support the hypothesis that catalytic RNA are relics of an ancient RNA world [38].

RNA has also been shown to possess a remarkable diversity of structural and metabolic functions. One of the most significant discoveries is that of riboswitches [39,40,41], RNA motifs that, by binding a small molecule ligand, can exert regulatory control over the transcript in a *cis*-acting manner. Riboswitches are now recognized as important and widespread elements in the control of gene expression in numerous evolutionarily distant Bacteria, Archaea, Plantae, Fungi and Algae [42]. Further evidence of the versatility and the functionality of the RNA is provided by the ribosome, which appears to be a ribozyme [43]. Indeed, ribosomal RNA (rRNA) occupies the central core of the ribosome and catalyzes peptide bond formation during protein biosynthesis [44,45,46], while the protein part of the ribosome plays a secondary role. The discovery of microRNAs (miRNAs) [47,48], a large family of small, approximately 21-nucleotide-long, non-coding RNAs, lends further support to the RNA world hypothesis. miRNAs are key post-transcriptional regulators of gene expression. In mammals, for instance, miRNAs were predicted to control the activity of approximately 30% of all protein-coding genes and were shown to participate in the regulation of almost every cellular process investigated so far [49]. Finally, the discovery of self-sustained RNA systems, which can catalyze their own replication, provides additional evidence that RNA is capable of functions presently performed by proteins [50,51,52,53].

However, there is still a missing piece of the puzzle in experimental models of RNA polymerization and replication, which would link the evolution of the products of non-enzymatic RNA polymerization to self-sustained RNA replicases. A major reason for this gap is the insufficient production of large oligomers and in high enough yields to adequately sample polymer structure and function. This obstacle has been partially addressed by recent reports on improved polymerization reactions [1,2,3], but functional RNAs remain elusive. In a complementary scenario, RNA and peptides may have mutually catalyzed the formation of one another since their earliest emergence [54,55], which would complete the puzzle by bypassing the problem of RNA self-replication.

## 4. Thermodynamics of RNA Polymerization

**Scheme 1:**
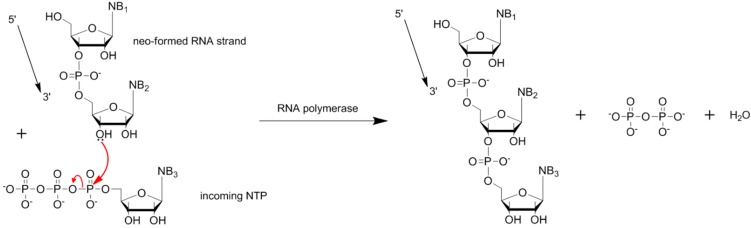
Phosphodiester bond formation in modern cell.

The synthesis of RNA under prebiotic conditions is a key step in chemical evolution. In modern organisms, nucleic acid polymers are synthesized from nucleotide triphosphates by polymerases, in the presence of magnesium ions as cofactors. Polymerases hydrolyze the nucleotide triphosphates into nucleotide monophosphates and pyrophosphates (phosphate ester dimer) and use the free energy released to drive the enzyme-catalyzed polymerization (Scheme 1). Prebiotically, in the absence of proteins, the polymerization of RNA could proceed via condensation between two monomers, where the hydroxyl group in either the 2’ or 3’ position of one monomer attacks the 5’-phosphorus of another monomer. A 2’,5’- or 3’,5’-phosphodiester bond is then formed, and a water molecule is released. This reaction is thermodynamically unfavorable in aqueous solutions, due to a positive Gibbs free energy (ΔG^0^ > 0). Moreover, most RNAs are unstable, being prone to hydrolysis at high temperatures or extreme pHs. A prerequisite of the RNA world hypothesis, however, is that early systems should have been robust to maintain evolution and transmission of genetic information. Thus, it has been proposed in the literature that RNA, like DNA, is also a product of evolution and that an “RNA-like” polymer was used by the earliest forms of life (reviewed in [56]). In the present work, we focus only on models of the synthesis of RNA oligomers under prebiotic conditions.

## 5. Chemical Activation of Mononucleotides

**Scheme 2:**
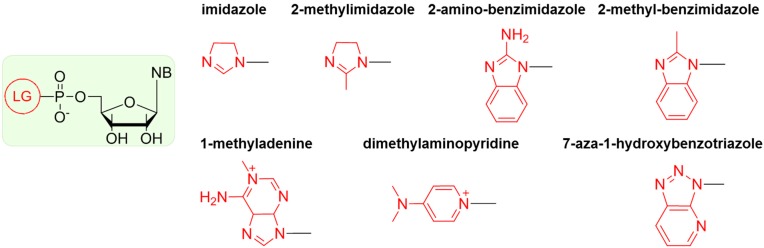
Structures of the most efficient leaving groups (LGs).

Attempts to oligomerize nucleotides in the absence of enzymes is a challenge; hence, nucleotides activated with various groups were synthesized to increase the rate of phosphodiester bond formation. These nucleotides are not utilized in modern organisms, but might have formed under primitive Earth conditions and promoted early RNA oligomerization [57,58,59,60,61,62,63]. An activated nucleotide is defined as a nucleotide in which the 5’-phosphate is bound to a nucleophilic group (generally, a nitrogen containing group) by an amide linkage and is ready “to leave”, thus releasing sufficient energy to drive polymerization. Derivatives of imidazole were particularly tested as leaving groups (LGs), because of their likely presence on early Earth in considerable concentrations [64]. The structures of the most efficient LGs reported in the literature are presented in Scheme 2.

**Scheme 3:**
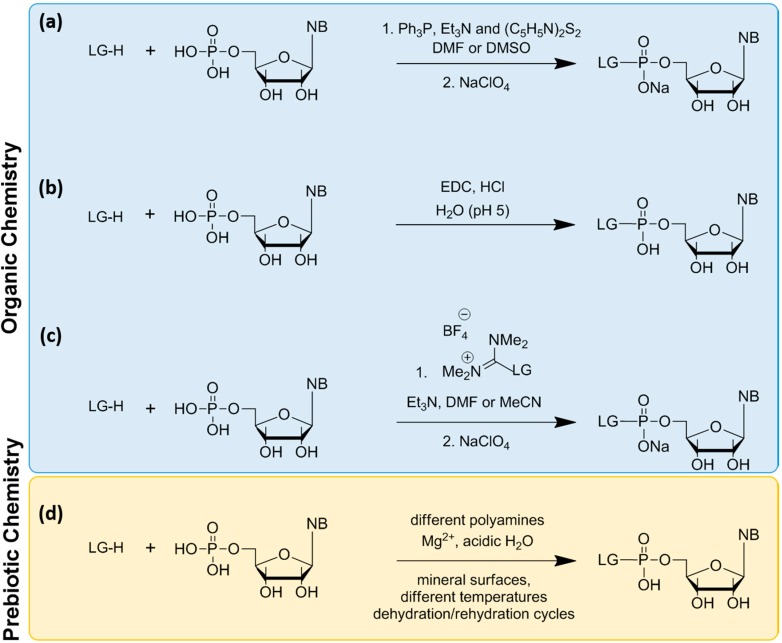
Summary of the methods used for chemical activation of nucleotides. (**a**) Mikayawa redox condensation; (**b**) activation with EDC in water; (**c**) activation with guanidinium salts; and (**d**) a proposed reaction involving polyamines and including mineral surfaces as potential catalyst, inspired from [59,65,76,77,78].

The synthesis of phosphoroamidates under prebiotic conditions with the use of different amino group containing molecules, such as urea, ammonia, imidazole, glycine, ethylenediamine, histamine, *etc.*, has been reported [59,65], but the yields were low (10%–50%, depending on the nucleotide). To overcome these low yields, three alternative methods were used: (a) the Mikayawa condensation reaction [63,66,67,68]; (b) activation with EDC (1-ethyl-3-[3-dimethylaminopropyl]carbodiimide hydrochloride) in water [69,70,71,72]; and (c) activation with uranium/guanidinium salts [25,73,74] (Scheme 3). However, these methods present many limitations. Activation through Methods (a) and (c) involves complex organic synthesis in organic solvents, and it is difficult to imagine a prebiotic environment with such conditions. Furthermore, a prerequisite for Method (c) is the synthesis of guanidinium salt of each LG, which is not always obvious [75]. Furthermore, the activated nucleotides are unstable and tend to hydrolyze with relatively short (~1 day) half-lives, while the oligomerization reaction occurs in several days. This problem necessitates feeding the reaction by washing out the spent nucleotides and replacing them with freshly activated nucleotides. Method (b) seems the most interesting, because the reaction occurs in aqueous solution and consists of a single-step, where the product can be used directly for oligomerization without the need for an intermediate purification step [25]. However, the oligomerization reaction still needs high amounts of carbodiimide, which was unlikely to be present on early Earth.

Mineral surfaces could have played a role in the activation reaction. For example, it was found recently that phosphate minerals, such as libethenite, Cu^2+^_2_(PO_4_)(OH), ludjibaite, Cu^2+^_5_(PO_4_)_2_(OH)_4, _reichenbachite, Cu^2+^_5_(PO_4_)_2_(OH)_4_, cornetite, Cu^2+^_3_(PO_4_)(OH)_3_, and hydroxylapatite, Ca_5_(PO_4_)_3_(OH), can promote nucleoside phosphorylation [77]. More interestingly, amorphous silica was also shown to promote phosphorylation [78]. These findings demonstrate the ability of mineral surfaces to catalyze the formation of high-energy compounds. Taking these ideas together, and using state-of-the-art analytical methods, it might be possible to identify novel prebiotic pathways to form phosphoroamidates or other reactive nucleotides (Scheme 3d). Varying conditions, such as temperature, pressure, hydration/rehydration and/or freeze/thaw cycles, could potentially provide the necessary energy to drive such reactions. In a major recent advance, the necessity for activating nucleotides by adding a leaving group was circumvented by a one-pot synthesis of cyclic monophosphate nucleotides [22,79,80,81]. The formation of such nucleotides is more plausible under prebiotic conditions compared to the activated nucleotides for many reasons. They are the preferred products of the prebiotic phosphorylation of nucleosides [82,83]; they are the natural products of RNA self-cleavage activity [84,85], and their polymerization in prebiotic conditions has been demonstrated [86,87].

## 6. Non-Enzymatic RNA Oligomerization

In this section, we describe four of the models that have been proposed in the literature for RNA oligomerization in the absence of a template.

### 6.1. Mineral-Catalyzed RNA Oligomerization

Minerals have long been hypothesized to play a key role in concentrating the products of prebiotic organic chemical processes leading to the origin of living systems [88]. The catalytic role of montmorillonite clay has been demonstrated in the polymerization of nucleic acids [19], peptides [20,89,90] and in the self-assembly of lipids [21,91]. In the case of RNA, non-enzymatic oligomerization was extensively and almost exclusively studied by the Ferris group for more than three decades. They have demonstrated that montmorillonite catalyzes oligomerization of activated nucleotides [92]. The oligomerization product can serve as a template for the synthesis of the complementary molecule regardless of the nature of phosphodiester linkages in the template [93]. Daily “feeding” with activated monomers induced the extension of a 10-mer primer up to 50-mer in 14 days [94], for both adenine (A) and uracil (U) nucleotides [95]. When 1-methyladenine is used as the activating group, 40–50 mers of A and U and 20–25 mers of cytosine (C) were formed in one day, even in the absence of a primer [96]. Regioselectivity in the phosphodiester bond formation was also reported [97,98]. The products of non-enzymatic oligomerization of RNA contained a mixture of 2’,5’ and 3’,5’ linkages. It was shown that 3’,5’-linked products were predominant if purine nucleotides were used, whereas 2’,5’-linked oligomers were mainly produced with pyrimidine nucleotides. However, regioselectivity may not be an obstacle to evolution, as was previously thought, and might even be an essential property allowing RNA polymers to emerge [99,100]. Although it is not in the scope of this review, another phenomenon, called regiospecificity, has been observed, when clays were involved in the oligomerization reaction in the presence of a template. In other words, the insertion of nucleotides in the *de novo* forming oligomer had a preference, in the order of A > G > C > U [97]. Regiospecificity is related to hydrogen bonding between the nucleotides and the template and to the stacking interactions between the nucleotides [101]. The effect of montmorillonite on the homochirality of the products has also been investigated. A racemic mixture of d,l-imidazolide of adenosine with d,l-imidazolide of uridine were reacted together in the presence of Na^+^-montmorillonite for three days. Homochiral products were formed, and their selectivity increased with increasing chain length of RNA oligomers up to pentamer [102,103]. The authors ascribed their observations to the presence of montmorillonite, although it was not clear whether the homochirality selection depends on the presence of montmorillonite or if it is just an intrinsic property of RNA.

#### 6.1.1. Effect of Salts, Temperature and pH

Since the discovery of the catalytic role of montmorillonite in RNA oligomerization [19], the standard physiochemical conditions for the reaction were 0.2 M NaCl, 0.075 M MgCl_2_ and pH 8 (0.1 M HEPES or PIPES buffer) at room temperature. A wider range of conditions was subsequently examined, including the effect of salts, temperature and pH [104]. The authors found that, while MgCl_2_ is the most efficient catalyst, some catalytic effect can be achieved by a high concentration (1 M) of NaCl, even in the absence of Mg^2+^. More recently, Joshi and Aldersley [105] investigated the role of metal ions and found that RNA chain length depends on the nature of the monovalent cation and anion with an increasing length in the order of Li^+^ > Na^+^ > K^+^ and Cl^−^ > Br^−^ > I^−^, respectively. Thus, LiCl resulted in a maximum detected length of 12-mers, while pentamers were the maximum length detected in the presence of KI.

Within a temperature range of 4–50 °C, the net rate of activated nucleotide hydrolysis *vs.* polymerization declined from octamers at 4 °C to hexamers at 50 °C [104]. Kawamura showed that, in aqueous solution at 75 °C, the rate of the phosphodiester bond formation rate is 50-times higher than its decomposition rate, and this trend can be extrapolated to temperatures over than 100 °C [106]. However, template-directed polymerization occurs poorly under these conditions (75–100 °C). To overcome this problem, Kawamura suggested the involvement of prebiotic enzymes or other molecules in promoting the association between the activated monomers and the template in a similar manner to what is known to occur in extant hyperthermophiles [107].

Oligomers can be obtained in a pH range of 6 to 10 with an optimum at pH 7–8 [104]. At pH 1–6, mainly diadenosine pyrophosphates (A^5’^ppA) were obtained, and activated nucleotides remained mostly intact, even after 50 days of incubation at pHs >10 [104]. These results were unexpected, because nucleotides are positively charged at low pHs and should adsorb better on the negatively-charged montmorillonite, thus yielding better oligomerization. Detailed studies of the role of montmorillonite were conducted in order to understand these results and to reveal the mechanism of RNA oligomerization on montmorillonite [108,109].

#### 6.1.2. Mechanism of Catalysis by Montmorillonite

**Scheme 4:**
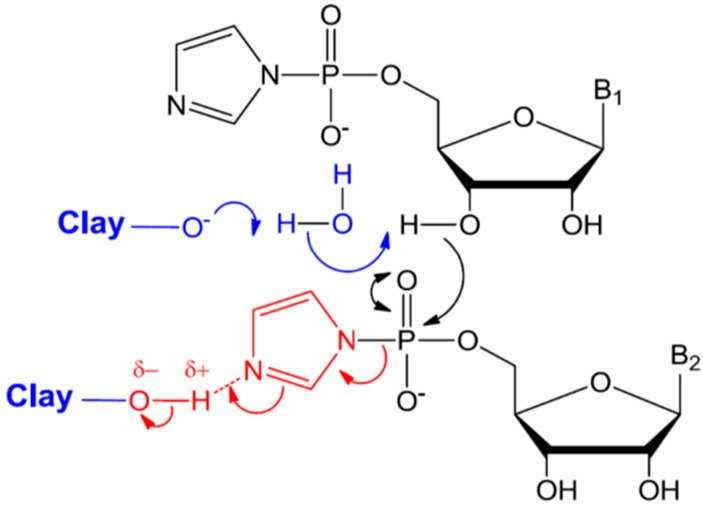
Proposed mechanism for phosphodiester bond formation on montmorillonite with general base/acid catalysis [111]. Reproduced here with permission.

A wide range of clay minerals and of varied provenance were investigated for their catalytic activity, but only a few montmorillonites were deemed excellent (generating heptamers or longer); and no apparent trend in clay structure or provenance was found [108,109]. In addition, only montmorillonites pretreated in a specific manner, which is different from the standard procedure, were found to be efficient catalysts. In geochemistry and soil chemistry experiments where clays are used, it is standard protocol to exchange the originally present interlayer cations by repeated washing of the clay with the desired cation solution. This helps to understand the role of a specific cation in the reaction mechanisms of interest. Ferris and co-workers found that clays pretreated by the standard procedure were not catalytic. Rather, only clays prepared by the Banin procedure were efficient catalysts [108]. In this procedure, interlayer cations are exchanged for protons by repeated washing with acid and subsequently back-titrating to pH 6–7 with base containing the desired cation [110]. Moreover, double the amount of alkali was needed for achieving the same pH in titration of non-catalytic montmorillonites compared to the catalytic one [108]. The difference between catalytic and non-catalytic montmorillonites was interpreted to be a result of differences in the stoichiometry of clays, resulting from isomorphous substitutions in their aluminosilicate framework during their original formation. These pH and ion dependence results are intriguing, because both the clay and the activated nucleotides are negatively charged at pH 7, where oligomerization was observed to be optimal [104]. To better understand this phenomenon, the authors investigated the pH-dependence of the adsorption and oligomerization of RNA on montmorillonite [111]. They found from adsorption isotherms that nucleotide adsorption increased with decreasing pH, consistent with the expectation that neutral or positively-charged activated nucleotides adsorbed on negatively-charged montmorillonite. However, oligomerization was still optimal at pH 6–8, where the nucleotides are monoanions or zwitterionic. Since the montmorillonite needed first to be treated by protons and then titrated to pH 7, the authors proposed a model in which the oligomerization reaction is catalyzed by both protons and the negatively-charged edge sites of the montmorillonite surface, through a general acid/base process. The negatively-charged edge sites of the montmorillonite serve as a base, enhancing the nucleophilic character of the hydroxyl group of the ribose, while the protons from solution are needed to stabilize the LG (Scheme 4). This model permits the authors to explain their previous irreconcilable sets of experimental results in a unified and consistent manner [109].

#### 6.1.3. Effect of Other Minerals

The potential effect of other minerals, such as olivine, galena, calcite, magnetite, rhodochrosite, sphalerite, magnesite, siderite, hematite, brucite, chalcocite, talc, dolomite, pyrrhotite, pyrite and the carbonaceous chondritic meteorites, Murchison, Yamato-791717 and Yamato-86751, were also tested. None of the tested surfaces was found to promote RNA oligomerization at rates better than or comparable to those of montmorillonite [104,112]. Even amongst the clays, it was found that Na^+^-, Li^+^- or Ca^2+^-montmorillonite are catalysts, but Mg^2+^-montmorillonite, smectite, nontronite, allophane, imogolite and sepiolite are not. However, other properties of the minerals, such as their bulk chemical composition, partial ionic substitutions, crystal structure, surface charge and particle size, should also be examined in detail. Interestingly, nucleotides were recently found to adsorb more strongly on nontronite than on montmorillonite and mainly at the edge sites, rather than the interlayer sites [113]; adsorption of nucleotides on the mineral surface, however, does not imply a relationship to their oligomerization efficiency. A further investigation in this direction would shed light on the specific site(s) of the oligomerization reaction, whether it is in the interlayer sites or on the edges.

### 6.2. Lipid-Catalyzed RNA Oligomerization

Models of prebiotic RNA oligomerization on surfaces other than clays have been reported. Rajamani *et al.* [23] demonstrated that lipids promote the formation of RNA-like polymers up to 100-mers from non-activated adenosine monophosphate (AMP) or uridine monophosphate (UMP). Based on a simple experimental design of hydration/dehydration cycles, a mixture of nucleotides and phospholipids was exposed to cycles of wetting and drying at a temperature ranging from 60 °C to 90 °C. At the end of the experiment, the lipids were extracted, and the RNA products were purified and analyzed by electrophoresis. No RNA oligomers were detected when the lipid was omitted from the reaction mixture, so it was concluded that the lipid plays a role in promoting the oligomerization by organizing and concentrating the monomers within its liquid crystal matrix. This conclusion was later supported by an X-ray scattering study, where the distance between the 5’-phosphate of AMP and the 3’-OH of the ribose was estimated to be only of ~2.1 Å, thus favoring the condensation reaction during dehydration [114]. The RNA-like molecule exhibited hyperchromicity (an increase of UV absorbance upon increasing temperature) and could be stained by an intercalating agent. Both results suggested the presence of secondary structures, including duplex species stabilized by hydrogen bonding [115]. In a separate study, it was shown that the nucleotide components played a complementary role, in which they bonded to and stabilized the self-assembly of prebiotic lipids. The presence of adenine (30 mM) at high salt conditions (300 mM NaCl) promoted the formation of decanoic acid/decanoate vesicles [116]. Together, the results of these two independent studies present a viable example of the mutualistic chemical evolution of prebiotic molecules toward increasingly complex molecular systems.

### 6.3. “Click Chemistry-Like” RNA Oligomerization in Water

Because it is still difficult to produce reasonably large yields of activated nucleotides in prebiotically plausible conditions, Di Mauro and coworkers chose to investigate the condensation of RNA cyclic nucleotides monophosphate (cNMP) as starting monomers in their model for non-enzymatic RNA oligomerization. 3’,5’-cGMP and 3’,5’-cAMP oligomerization yielded 25-mers and up to octamers of RNA molecules, respectively. Other cNMPs, such as 3’,5’-cUMP, 3’,5’-cCMP or 2’,3’-cAMP, yielded only short oligomers not longer than pentamers [117]. The conditions at which the oligomers were formed were the simplest among all of those tested in the literature. The addition of formamide, the presence of copper phosphate minerals [77] or the addition of sodium pyrophosphate or sodium triphosphate did not have a significant effect on the lengths or the yields of the produced oligomers. The oligomerization reaction occurred in water at temperatures ranging from 60 °C to 90 °C with an optimum at 85 °C. Oligomerization of 3’,5’-cGMP was tested at concentrations ranging from 1 µM up to 100 mM, and the optimum concentration was found to be 1 mM. The reaction displayed biphasic kinetics, suggesting a two-step mechanism, in which oligomers were rapidly formed from monomers in the first step, followed by ligation of short oligomers [118,119,120], thus resulting in polymers up to 100-mers in 200 h.

**Scheme 5:**
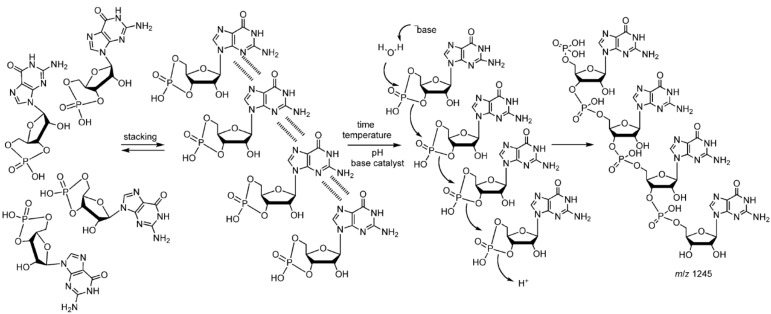
Proposed mechanism of polymerization of 3’,5’-cGMP [122]. Reproduced here with permission.

Subsequently, the authors proposed a molecular reaction pathway (Scheme 5), involving three major steps: (1) stacking of monomers; (2) nucleophilic ring opening of the cyclic phosphate group; and (3) “click-like” chemistry, leading to the formation of the 3’,5’-chain of RNA. Click chemistry refers to a reaction joining small modular units, with high yields, in a stereospecific way, and under simple reaction conditions, including a benign solvent, preferably water [121]. Lewis or general bases were shown to favor the reaction. The use of the sodium form of the cyclic nucleotides instead of its free acid form inhibited oligomerization, most likely because accessibility of the phosphate group was hindered by the interactions with the sodium ions [122].

**Figure 3:**
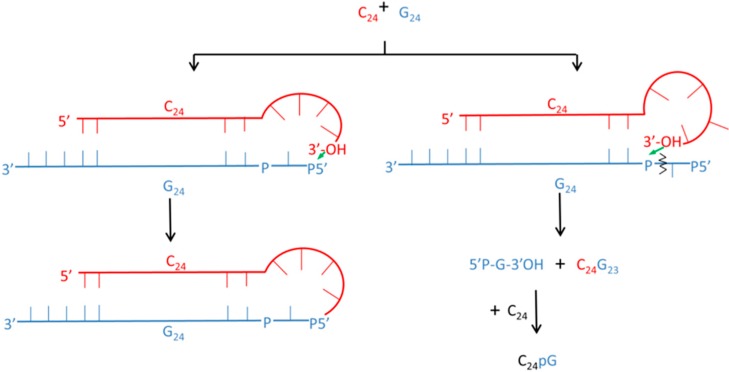
A mechanism of ligation following an intermolecular cleavage. The reaction between C_24_ and 5’-phosphorylated G_24_ is shown as an example. On the left: ligation assuming loop formation at the 3’-end of C_24_ and attack at the phosphorylated 5’-end of G24, leading to the formation of C_24_G_24_. On the right: simultaneous cleavage reaction initiated by the attack of the 3’-end of C_24_ at the last-but-one phosphate of the 5’-phosphorylated G_24_. The products of this reaction are C_24_G_23_ and 5’-phosphorylated guanosine-phosphate, which combines with C_24_, leading to the formation of C_24_pG. Reproduced here with permission [125].

RNA products of this system were shown to possess functional activity. The neo-formed oligo-G, which is the product of 3’,5’-cGMP oligomerization, could act as a ribozyme, which can self-ligate to its complementary RNA strand (oligo-C) through two routes (Figure 3). The discovery of these different routes for ligation suggests an early recombination mechanism between complementary sequences, which increases the complexity of the RNA repertory for early evolution towards the RNA world.

In an attempt to further explore the extremely fast initial rates of oligomerization, the reaction of cGMP was considered by another group [123]. It was found that oligomerization of 3’,5’-cGMP is more efficient under near dry conditions than in water. This conclusion is interesting, because it converges with the lipid-catalyzed oligomerization model and also with another model from the early 1970s, where oligomerization of 2’,3’-cAMP occurred in the dry state at moderate temperatures (25–85 °C) [86].

The availability of cyclic monomers in prebiotic conditions is not impossible [77,124]. Additionally, 2’,3’-cNMPs are the natural products of RNA degradation and possess several significant and advantageous properties that make them particularly viable substrates for prebiotic RNA synthesis [85].

### 6.4. Peptide-Catalyzed RNA Oligomerization in Eutectic Phase

Peptide-catalyzed RNA oligomerization in the water-ice eutectic phase was also found to be feasible. The eutectic phase of water-ice occurs at a temperature of −18 °C, where the majority of the solvent is transformed to the solid pure-water phase. The remaining part of the solution is liquid and contains the solutes at a much higher concentration. This particular environment could have led to the concentration of the monomers out of the bulk aqueous solution, thus favoring their oligomerization. The idea of such an environment emerged in the early 1990s [126,127] and was first proved by Kanavarioti *et al.* [128], who showed that uridine 5’-monophosphate imidazolide, which is the most challenging nucleotide to oligomerize [97], can form oligomers up to 11-mers in the presence of Mg(NO_3_)_2_ and Pb(NO_3_)_2_ at pH 6.9 in 31 days at −18 °C. This model of a microenvironment is supported by theoretical arguments and experimental results. For instance, some investigators have argued that much of the water on early Earth was frozen, but would have undergone periodic thaw/freeze cycles, due to bolide impacts or volcanic activity [12,129,130,131]. Others have proven that adenine, amino acids and pyrimidines can be synthesized in frozen aqueous solution [132,133]. Furthermore, a truncated hairpin ribozyme was found to restore its ligation activity at freezing temperatures, and this activity was promoted by freeze/thaw cycling [134]. All of these results support a cold site for the origins of life. However, the physical environment(s) that energized the first prebiotic processes is still largely debatable.

The eutectic-phase model may support the concept of synergism in the emergence of life. A dipeptide, serine-histidine (SerHis), was found to catalyze the formation of RNA phosphodiester bond in guanosine 5’-monophosphate imidazolide in the water-ice eutectic phase at pH 6.5 over 30 days at −18 °C [24]. Although the yield and length of the products are significantly lower compared to the other models detailed above, this study provided experimental support for an early co-evolution of prebiotic RNA oligomers and peptides, which would have given rise to a very complex repertory of prebiotic molecules. In other words, an organic catalyst could have emerged and evolved at the very first stages of RNA oligomerization. SerHis is only one example of a possible “primitive organocatalyst”. In order to find other possible primitive organocatalysts with better efficiency and to understand the structure-function relationships of primitive organocatalysis, a new concept based on a chemical synthetic biology approach was recently proposed [135].

### 6.5. Discussion of the Results of Non-Enzymatic RNA Oligomerization

Four among the numerous chemical models that have been proposed to simulate prebiotic synthetic pathways towards informational RNA oligomers were presented here. These models represent significant advances in non-enzymatic RNA oligomerization, especially when long oligomers were obtained. However, several limitations still exist and bear further investigations. In the mineral-catalyzed model, clay surfaces were used to concentrate and protect organic compounds of the primitive soup, but plausible monomers capable of undergoing oligomerization are still a challenge. The temperature is also a critical parameter that needs to be re-thought. Early Earth’s temperatures are assumed to be extreme compared to current Earth’s temperatures. For example, temperatures were either high (80–110 °C), an assumption supported by ^18^O isotope analysis of the oldest mineral known [14] and by models of the prebiotic Earth atmosphere [136], or very high (150–250 °C), if life had emerged near hydrothermal submarine vents [137]. The temperature could also have been very low (−20–0 °C) according to an alternative hypothesis for the site of the emergence of life [12,129,131]. The lipid-assisted RNA polymerization model is of particular interest for many reasons. First, as with extant life, activation of nucleotides is not required. The energy driving the polymerization reaction was provided by heat and cycles of hydration/dehydration. These cycles simulate environments of geothermal fields, such as hot springs and fumaroles, which were likely to have been common on early Earth. Third, the reaction was faster if dehydration was carried out using a stream of carbon dioxide or nitrogen, but not in air. This finding is consistent with the reduced atmosphere of early Earth. An interesting observation is that the oligomerization reaction occurred at acidic pHs (2.2–6.8) in the absence of any buffer or ion, in contrast with the clay-catalyzed model. However, a complete characterization of the product by analytical methods, such as mass spectrometry, should be undertaken as a next step. The model of cyclic nucleotide oligomerization in water is also interesting, because it was found to be robust under a range of environmental conditions. Although a more recent study was unable to reproduce all of the results in aqueous phases [123], it was demonstrated that substantial polymerization of cGMP did occur if the solution were dried. Peptide-catalyzed oligomerization is a very promising model and bears further investigation. However, synergism was not explored.

## 7. A Proposed Experimental Model

In the models reviewed above, it was shown that mineral surfaces, lipid surfaces and peptides can separately promote RNA oligomerization. Although it is very challenging and speculative to mimic a prebiotic environment, it is possible to arrive at a new model combining features of the previous studies. It is more logical to consider the co-evolution of all of these building blocks of life in one system. Other prebiotic molecules, such as aliphatic diamines, imidazole, nucleic acid bases, *etc.*, should also be included in the experimental system. Indeed, the more heterogeneous the soup, the more prebiotically plausible it is. Technically, this will lead us to a humongous number of starting conditions, which is very difficult to simulate in the laboratory, but it would be an improved approach.

Here, we propose a quaternary system consisting of mononucleotides, amino acids, montmorillonite and single-chain amphiphiles, in which potential synergism between the catalysts and mutualism between RNA and peptides are anticipated in their co-emergence and co-evolution. This model recapitulates the RNA-peptide world [54] and can be tested experimentally. In brief, the quaternary system is subjected to cycles of environmental change to promote polymerization. Encapsulation in vesicles and size exclusion is subsequently used to separate the protocells. The protocells are then lysed, and the RNA and peptides products are extracted and recycled with fresh reactants and catalysts. The expectation is the formation of complex polymers with structure and functionality as a result of synergism between different catalysts, mutualism between RNA oligomers and peptides and self-recognition/self-assembly processes of all of the biomolecules. The detailed experimental development of this conceptual model will be presented in a subsequent paper.

## 8. Concluding Remarks

The RNA world hypothesis is widely believed to be the best available hypothesis for the emergence of life on Earth. However, there is still a gap between the products of non-enzymatic RNA polymerization and the self-sustained RNA replicases, due in part to the lack of consideration of feedback processes. To bridge this gap, a complementary hypothesis related to the RNA-peptide world was highlighted herein. In this hypothesis, RNA and peptides might have emerged and evolved simultaneously and probably in a mutualistic manner. RNA products would have played the role of the ribosome, and catalytic peptides would have been prototypes of the current polymerases. Finally, the emergence of functional RNA would have preceded an accurate replication of RNA. To test this hypothesis, we have outlined briefly an experimental model where different catalysts may interact synergistically to enhance RNA polymerization. Re-incubation of RNA and peptide products in a fresh soup could lead to their mutualistic interaction. If isolation of functional RNA from the pool of produced oligomers could succeed, such a model would contribute to determining one missing piece of the puzzle of the origins of life.
